# Why Do Students Walk or Cycle for Transportation? Perceived Study Environment and Psychological Determinants as Predictors of Active Transportation by University Students

**DOI:** 10.3390/ijerph18041390

**Published:** 2021-02-03

**Authors:** Monika Teuber, Gorden Sudeck

**Affiliations:** Institute of Sports Science, University of Tübingen, 72074 Tübingen, Germany; gorden.sudeck@uni-tuebingen.de

**Keywords:** active transportation, physical activity, perceived study environment, psychological determinants, motivators, barriers, university students, socio-ecological approaches

## Abstract

University students are particularly at risk to suffer from physical and psychological complaints and for not fulfilling health-oriented physical activity (PA) recommendations. Since PA is linked with various benefits for health and educational outcomes, the group of students is of particular interest for PA promotion. Although active commuting has been identified as a relevant domain of PA in order to gain the various benefits of PA, little knowledge is available with respect to university students. This study tested conditions in the study environment, as well as personal motivators and barriers, as determinants for the active transportation of university students. Using a cross-sectional convenience sample of a university in the southwest of Germany (*n* = 997), we applied factor analyses to bundle relevant information on environmental and psychological determinants (adapted NEWS-G; adapted transport-related items from an Australian university survey) and blockwise hierarchical regressions. The objective was to analyze associations between the bundled determinants and self-reports on PA for transport-related walking and cycling (measured by the EHIS-PAQ). Results revealed associations between transport-related cycling and the perceived study environment (e.g., high automobile traffic) as well as certain personal motivators and barriers (e.g., time effort or weather conditions). The study contributes to the knowledge about determinants that are important for the development and improvement of public health interventions for students in a university setting.

## 1. Introduction

Academic studies often impose high demands on university students, which can be associated with negative effects on health. Students suffer more often from perceived stress [1] and from physical and psychological complaints than their peers [1,2,3,4]. As health is positively related with physical activity (PA) and less sedentary behavior, these behaviors can provide starting points for improving the students’ health: because students who are more physically active through sports or everyday activities have fewer complaints and a greater sense of well-being than inactive students [2,4,5,6]. For the same reason, active transportation is associated with less obesity, less cardiovascular risk factors, and higher physical fitness for students [7,8].

Since the transition from school to university often marks a particular risk for becoming physically inactive [9], the group of students is of particular interest for PA promotion in order to gain health benefits. According to current guidelines for health-enhancing PA, about half of the students in the United States, Canada, and China, 40% in Australia, and 67% in Europe are not sufficiently physically active [10]. Reasons for students’ physical inactivity are increasing self-employment, increasing academic workload with resulting problems in time management regarding work and social demands [8], and an increasing distance from home to university [11].

To counteract this, the promotion of PA in university settings is necessary. Due to the increasing number of people who will study, universities have a growing potential to reach a large mass of young adults in order to promote positive PA behavior, which will last in later life. However, in contrast to school settings, the promotion of PA is not yet widespread in university settings, which leads to a gap between school-based and workplace-oriented approaches of PA promotion. Moreover, the knowledge about determinants of PA in university students is scarce, but this knowledge is necessary to guide evidence-based PA promotion in university settings [12].

**Figure 1:**
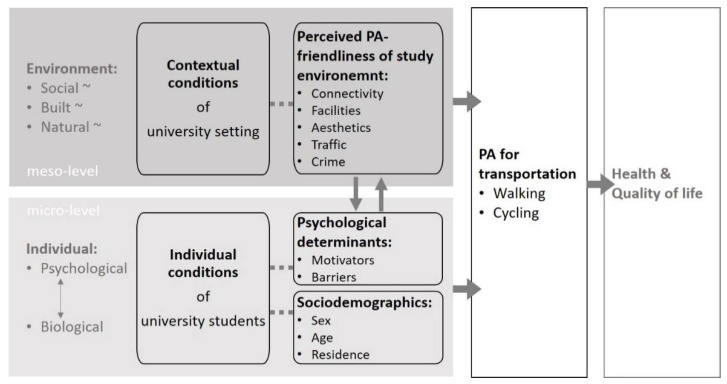
Schematic representation of the socio-ecological approach of PA promotion adapted to the university setting (own presentation based on Bucksch et al. (2012) [13] and Bauman et al. (2012) [12]).

Since the 2000s, PA promotion research has emphasized that the physical and social environment play an important role for PA behavior. Socio-ecological approaches increasingly have taken this into account and complement individually-focused approaches [12]. For example, Bauman and colleagues [12] as well as Bucksch and colleagues [13] differ between personal/individual and contextual/environmental factors that contribute to differences in PA behaviors. According to these basic ideas, Figure 1 schematically depicts individual and contextual factors of students’ PA behavior which are important to understand in order to develop and improve interventions for active transportation, which can lead to an higher level of physical activity and in turn to a better health status [12]. Adapted to the university setting, the perspective of students’ individual conditions is integrated into the perspective of the surrounding conditions of the study environment, increasing the extent of the effect radius of the PA promotion when regarded together [14,15,16,17]. Hence, this adaption follows public health and socio-ecological approaches [13,15,18].

Some empirical studies in the university setting already exist, which have revealed several factors important for the active transportation behavior of students [19,20,21,22,23,24,25,26,27,28,29,30]. The results show basically that encouraging students to commute to university by bicycle or by foot is linked with the learning environment as well as with the campus environment, which deliver more or less activity-friendly physical environments. 

The connectivity of the street network has been identified as an important determinant for the cycling behavior of students [22,24,27,28,30]. This refers, for example, to intersection density [28], street connectivity [24], and bicycle racks installed on buses to extend the commuting distance [20]. Such improvements to the cycling infrastructure reduce effort and time demands, which in turn mitigate the negative impact of distance [21,22,26,28,29] and increase the likelihood of cycling for commuting reasons [30].

In addition, the availability and proximity of walking or cycling facilities encourage students to cycle more [19,20,21,23,25,27,28,30]. However, also in terms of active commuting in general, the perception of walking and cycling facilities are positively associated with active commuting to university [23].

The feeling of safety also contributes to increased active transportation of students. Traffic safety, for example, based on traffic-calming measures [22], has been shown to be important for the active commuting of students [19,22,27]. On the other hand, safety concerns can lead to avoidance of active commuting. Such is the case, for example, with high automobile traffic including sharing the roadway with automobile traffic [19]. Moreover, crime issues are related to students’ active transportation behavior [21,25,27,28]; this refers to personal safety as well as to bicycle security such as secure bicycle-parking racks and lockers, and a high degree of safety against bicycle theft [22,25,27].

Finally, there are the aesthetic aspects, which are positively related to active transportation and are expressed, for example, by the “attractiveness of the surroundings” [27] (p. 72).

In addition to environmental conditions, potential personal motivators, and barriers among students’ active forms of transportation are also known from empirical studies [19,20,22,23,25,26,28]. For example, motivators such as concerns for the environment increase the probability of choosing bicycles [28]. Barriers such as travel costs [26,28] or inclement weather [25] prevent students from active transportation. In addition to the barrier of time effort [26], there are other types of effort that prevent students from active transportation such as planning [23], inconvenience, time constraints [21], or physiological discomfort [27].

The current state of research leaves open questions regarding the environmental and personal determinants of active transportation behavior in university settings. So far, only a few studies have dealt with such questions by considering environmental and psychological determinants together. Especially the environmental variables have been less studied, but are thought to have widespread effects for active transportation behavior [12]. Furthermore, there is less known about the differentiation in various modes of active transportation, as most often only general PA or a specific mode of transportation is considered. In addition, there is a lack of consistent measurement methods. Since the relationship of the environment to physically active behavior has also been studied in the community neighborhood, various survey instruments have been established in the communities for assessing the neighborhood environment [17,18,31,32,33,34,35,36,37,38,39,40,41]. None are yet available for the study environment. Therefore, there is a lack of both a general more extensive survey procedure of the PA-friendliness of the study environment and investigations on how this relates to the two transport-related modes of PA, walking and cycling.

## 2. Purpose of the Study

The present study addresses the question of which conditions of the study environment as well as personal motivators and barriers are related to the active transportation behavior of university students. Relationships are considered separately for transport-related walking and cycling because different modes of transportation can have different interactions with the environment.

This question is addressed because students are particularly at risk of not fulfilling health-oriented PA recommendations and active transportation has been shown to be a relevant domain of PA that brings various health-promoting benefits of PA. As students suffer from physical and psychological complaints, promoting active transportation could counteract this. This requires specific knowledge of socio-ecological determinants. So far only a few studies have considered both personal and environmental determinants together [22,23,27].

By this study, additional specific knowledge about why students walk or cycle will be gained to support the implementation of specific interventions in the university setting to improve personal health and, beyond that, public health.

To achieve the purpose of the study, two steps are carried out. Since there is no established survey instrument available for the study environment, in a first step the questionnaire for the Neighborhood Environment Walkability Scale for Germany (NEWS-G) was adapted to the study environment. NEWS-G offers a comprehensive collection of environmental characteristics across several sub-chapters and is one of the most widespread measurement procedures of the perceived PA environment [31]. The adapted version should represent a coherent construct for friendliness of the study environment regarding transport-related PA. For the individual perspective, motivators/barriers that are interrelated will be exploratively clustered so that the relationships between them are considered. In the second step, regression analyses will be conducted to empirically identify associations between individual as well as environmental factors and the two separate outcomes of transport-related walking and cycling in terms of health-enhancing activity. We assume that, in the context of the socio-economic approach, both psychological and environmental factors are related to the active transportation behavior of university students.

## 3. Materials and Methods

### 3.1. Design, Setting, and Sample

The cross-sectional convenience sample for this study (*n* = 997) was formed by students at the University of Tübingen in southwestern Germany who completed the survey. The University of Tübingen represents an urban university setting integrated into an urban hilly landscape. It consists of eight faculties plus a further five interfaculty institutes. More than 200 courses of study are offered. The cross-sectional study was conducted as part of the PA promoting project “BeTa*Balance*” of the university sports organization at the Institute of Sports Science of the University of Tübingen. The study was performed online for three weeks during the end of the university time of the summer semester in 2018 and addressed all students at the university. The online questionnaire was distributed via the university’s mailing list and Facebook posts, as well as outreach campaigns in cafeterias and at university meeting points (e.g., the University library and lecture halls) using flyers with a QR code leading to the link for the online questionnaire. The Ethics Commission of the Faculty of Economics and Social Sciences at the University of Tübingen gave a positive vote for the study procedures.

Of a total of 999 returned questionnaires, 997 form the sample of this study, since two cases were not usable due to incomplete answers. Among the participants were 718 female students (72%), 232 male students (23.3%), and 47 (4.7%) did not provide any gender information. The average age of the surveyed students was 23.4 years (SD = 3.45), with an age range from 18 to 42 years. A total of 224 students (22.5%) of the sample reported that they are not living in town and commute to the university town (Table 1). In total, 26,151 students were registered in the summer semester 2018 of the University of Tübingen (state 15 May 2018), resulting in a response rate of 3.8%. Of these, 58.2% were female and 42.8% were male. The sample in this study showed a shift toward more female students.

### 3.2. Measures

#### 3.2.1. Physical Activity: Transport-Related Walking and Cycling

To assess PA, the instrument from the European Health Interview Survey (EHIS-PAQ) was used, which records domain-specific information on PA for transport-related walking and transport-related cycling [42]. The questionnaire enables the determination of activity volume in different activity domains. In the domain of active transportation, the participants answered the following questions, each worded in the same way, but separately for walking and cycling in relation to a typical week: (a) “In a typical week, on how many days do you walk/bicycle for at least 10 min continuously to get to and from places?” and (b) “How much time do you spend walking/bicycling in order to get to and from places on a typical day?” Activity volumes were determined in accordance with the procedure in the validation studies [42] and were subsequently indicated as duration per week (minutes or hours). Time values were transformed in metabolic equivalent (MET) values using 3.3 as a factor for computing MET-minutes for walking and 6.0 for cycling, which corresponds to the procedure in the validation studies [42]. As a guideline, 1 MET equals the energy expenditure in the state of complete rest [43].

#### 3.2.2. Contextual Conditions: Perceived PA-Friendliness of the Study Environment

For the assessment of the perceived study environment, the German version of the Neighborhood Environment Walkability Scale (NEWS-G) [44] was contextualized to the university setting. Thus, it can be applied to everyday study life and it records the PA friendliness of the study environment. The following two sections were relevant for this article: (1) opportunities for walking and cycling (including land use mix–access, street connectivity, walking/cycling facilities, and environmental design) and (2) (traffic) safety (including crime) (Appendix A Table A1). Both areas consider relevant factors of leisure-related resources, appearance, and land use, and they are also congruent with the categories of the instrument “Neighborhood Active Living Potential” [45]. Both sections of the adapted version of NEWS-G consist of different statements for which the participants had to indicate their degree of agreement: 1 = totally disagree; 2 = more likely to disagree; 3 = more likely to agree; 4 = totally agree. For the purpose of the main analysis, the items of the study environment were bundled to main factors in the pre-analysis to get a dimensionally reduced yet statistically coherent measurement of the perceived PA-friendliness of the study environment (see Section 4.1.1).

#### 3.2.3. Individual Conditions: Psychological Determinants of Active Transportation—Motivators and Barriers

The survey instrument of Shannon et al. (2006) guided the measurement of motivators and barriers. This instrument was used in a study with university students in order to analyze motivators and barriers for active commuting [26]. All items include statements, which should be rated according to the extent to which they either motivate or prevent one toward or from engaging in active transportation behavior (e.g., Mot1, “Potential to save money”, or Bar1, “Inappropriate weather” with the answer choices: 1 = not at all, 2 = a little, 3 = strong, 4 = very strong). There was also an option, "I cannot judge", which was included in this study for further analysis as an item-nonresponse, since a simultaneous increase in the proportion of people who claim to have no opinion does not allow for the attribution of an actual lack of opinion [46,47,48].

In addition, two items were added (Mot5, and Mot8) which describe study-related motivator-items. Both were supplemented by the reasons for doing sports or PA, which were asked in the questionnaire for the study “GEDA 2014/2015-EHIS” [49] (p. 118) (Appendix A Table A2).

For barriers, three items were added which should account for the hilly conditions of the university setting (Bar2), study-related barriers (Bar5), and barriers relating to mood or desire (Bar12). Here, concerns of “GEDA 2014/2015-EHIS” [49] (p. 118) and the study by Krämer and Fuchs [50] (p. 174) were used to complement the specific areas (Appendix A Table A3).

Again, factor analyses were applied to obtain dimensionally reduced data on barriers and motivators for active transportation. This was done in the pre-analysis in order to bundle relevant information to be considered later in the main analysis (see Section 4.1.2).

### 3.3. Statistical Analysis

In a first step (pre-analyses), exploratory factor analyses (EFA) were conducted separately for the items measuring the study environment as well as for the psychological determinants of active transportation (in terms of motivators and barriers). Therefore, the IBM SPSS 25 software package was used. The decision regarding the number of factors extracted was based on both statistical indices (eigenvalue scree plot, commonalities *h*^2^, factor loadings, and internal consistency of bundled items *r/α*) as well as content-related fit with the literature and the dimensions measured by the NEWS-G.

In a second step (main analyses), blockwise hierarchical regression analyses using IBM SPSS AMOS 25 software was applied. This was done in order to analyze associations between self-reports on PA, the perceived study environment, and psychological determinants of active transportation. Dependent variables were, separately, transport-related walking (A) and cycling (B). Firstly, in the blockwise procedure, sociodemographic factors were included as predictors (sex, age, and whether or not a resident in the university town). Secondly, determinants of the perceived study environment were included, and thirdly the psychological determinants of motivators and barriers were added as predictors in the regression model.

Missing values were estimated in the main analysis using the full information maximum likelihood (FIML) method implemented in AMOS 25. This was done in the case when at least one item of a scale measure was missing. In those cases, no mean value was calculated for the respective scale measure that could be included in the main analysis. Accordingly, the number of cases *n* for certain scales was reduced due to missing scale mean values.

For the evaluation of the global model fit, the root mean square error of approximation (RMSEA) [51], the comparative fit index (CFI) [52], and the minimum of discrepancy in relation to the degrees of freedom (CMIN/DF) were used [51,52]. In order to compare the different models within the hierarchical blockwise approach, information on the determination of variance (R^2^) and the change of R^2^ compared to the previous model were calculated.

Furthermore, the regression models were specified in a way that included significant correlations between the predictors. This led to an acceptable and good model fit, which ensures the model-based estimation of missing values. In addition to tests for statistical significance (*α* < 0.05), effect sizes were determined and interpreted—according to small effects in agreement with Cohen (1988) [53]—if the standardized regression coefficient is equal to or higher than *β* ≥ 0.10.

## 4. Results

### 4.1. Pre-Analyses: Exploratory Factor Analyses

#### 4.1.1. Contextual Conditions: Perceived PA-Friendliness of the Study Environment

The bundling of the 14 initial items for the perceived study environment resulted in a differentiation of seven factors. A comparison of the finally derived factors and their content fit with the categories of the NEWS-G [44] and were adapted for the study environment as reported in Appendix A (Table A1). In this bundling process, we considered the subsequent categories of the NEWS-G: (C) “Land use mix–access”, (D) “Street connectivity”, (E) “Walking/Cycling facilities”, (F) “Aesthetics”, (G) “Pedestrian/automobile traffic safety”, (H) “Crime safety”. The EFA with 14 items initially proposed a five-factor solution, however, the results of the EFA led to the elimination of two items in order to improve the reliability of the factor regarding traffic safety. This concerns two items of category G, pedestrian/automobile traffic safety (Item G3, “The traffic speed in most surrounding streets is normally low (30 km/h or less)”, and item G6, “There are crosswalks and pedestrian signals to help walkers cross busy streets in my study environment”). The item wordings, item descriptives, factor loadings, and communalities are reported in Appendix A (Table A4).

For the remaining items, statistical- and content-based criteria indicated a preference for a more differentiated seven-factor solution after keeping individual items that could not be bundled well to one factor, but which contributed to a variance explanation with reasonable communalities. They were either included as a single item in the further analyses after a content-wise comparison with the categories of the NEWS-G, or were assigned to other content-wise suitable factors after statistical verification by reliability analyses. The derived seven factors reflect the following areas of the perceived PA-friendliness of the study environment:

- The factor, “active transportation: walking/cycling facilities”, contains two items (E1, and E3), both of which originally belonged to the category E “Walking/cycling facilities” of the NEWS-G. They combine aspects of available sidewalks and the proximity of bicycle or pedestrian trails. They showed a significant inter-item-correlation (*r* = 0.51).
- The factor, “aesthetics”, comprises three items, which refer to the corresponding category of the NEWS-G (F1, F3, and F5). They refer to trees along the streets, interesting things to look at, and a lot of nature in the study environment. The internal consistency of the factor was rather low, but still satisfactory with respect to group-based analyses (Cronbach’s *α* = 0.60).
- The factor, “high automobile traffic”, bundles three items that represent the difficulty, unpleasantness, or insecure feeling when walking/cycling due to much traffic and the noticeable exhaust fumes from cars or buses. This factor showed a satisfying internal consistency (Cronbach’s *α* = 0.69).
- “General crime” is presented by a single item, which describes the unsafe feeling during the day due to crime (New2). It was separated from another crime-related item.
- “Bicycle-related crime” is represented by a single item (New3). This item describes the unsafe feeling of leaving even a locked bicycle in the study environment.
- The factor, “active transportation: uphill”, is reflected by one single item which describes the limited number of routes for getting from place to place due to a hilly landscape (C7). It was the only item that covers the category C “Land use mix–access” from the NEWS-G.
- The factor, “active transportation: connectivity”, is described by one single item which stands for alternative (walking/biking) routes for getting from place to place (D5). It originally belonged to the category D “Street connectivity” from the NEWS-G.

#### 4.1.2. Individual Conditions: Psychological Determinants of Active Transportation—Motivators and Barriers

The analyses to bundle psychological determinants of active transportation resulted in a differentiation of four factors for motivators and three factors for barriers. To arrive at these results, the following steps were taken. The EFA for motivators suggested the formation of two factors, after having previously removed item Mot6 (opportunity to socialize) due to a very low communality value (*h*^2^ = 0.23). Mainly based on content-related considerations as well as on information of internal consistency statistics, and inter-item-correlations, we decided to split up two factors and preferred a four-factor solution. The item wordings, item descriptives, factor loadings, and communalities of motivators are reported in Appendix A (Table A5).

- The topic, “comfort with study life”, includes two items, which describe the motivation of active transportation because of being more efficient for study and work and because of the active balance between and after courses. The items showed a high inter-item-correlation (*r* = 0.53).
- The factor, “personal benefits”, comprises two items with motivators such as joy, health, and fitness. According to the results of the EFA, they belong to the same factor as the study-related items. As mentioned above, we preferred to separate the study-related items and other personal benefits in order to be able to be more specific regarding the university setting. The items showed a satisfactory inter-item-correlation (*r* = 0.43).
- The label, “instrumental extrinsic benefits”, summarizes two items regarding the potential to save money and to avoid the search for a parking space. These items showed a satisfactory inter-item correlation of *r* = 0.43.
- The topic, “avoid air pollution”, is reflected by one single item that was separated from the former extrinsic benefits factor. It revealed the lowest factor loading (λ = 0.48) and did not fit to the content of the other instrumental extrinsic benefits.

The EFA for barriers to PA suggested using three factors, after having previously removed two items due to overlap with items of the perceived study environment regarding the hilly landscape and the lack of secure bicycle parking facilities. Regarding the results of the EFA, two more items were excluded that could not be satisfactorily assigned to one factor due to statistical reasons (Bar3, and Bar10). Additionally, item Bar10, which refers to the lack of knowledge of the quickest and easy routes, had the lowest communality (*h*^2^ = 0.34). The item wordings, item descriptives, factor loadings, and communalities are reported in Appendix A (Table A6). For the remaining items, the following three factors were considered:

- Factor, “discomfort with study life” (Cronbach’s *α* = 0.77), consists of three items that describe barriers related to everyday life at university such as an uncomfortable feeling participating in university courses after physical exertion, the necessity of bringing a change of clothes, or the lack of or poor changing/shower facilities (Bar5, Bar6, and Bar7).
- The factor, “personal barriers” (Cronbach’s *α* = 0.69), summarizes three items which describe barriers of physical effort, time effort, and bad mood (Bar4, Bar11, and Bar12).
- The factor, “external barriers” (Cronbach’s *α* = 0.65), comprises two items which describe barriers referring to the weather conditions and time of day (Bar1 and Bar2).

Table A2 and Table A3 in Appendix A summarize the results regarding the motivators and barriers for PA. Moreover, Table 1 gives an overview for the finally considered determinants of active transportation behavior and provides descriptive information.

### 4.2. Main Results: Regression Models

The main analysis consisted of two separate analyses for the respective dependent variables of transport-related walking (A) and transport-related cycling (B). For each type of transportation mode, bivariate correlations and a separate regression model were calculated. In the basis model, we included the three sociodemographic indicators (sex, age, resident in university town; Model A0 and Model B0). We then added blockwise the indicators of the perceived study environment (models A1 and B1) and the indicators for personal motivators and barriers (models A2 and B2) (see Table 2 and Table 3).

#### 4.2.1. Regression Analyses for Walking

The regression models for active transportation by walking showed good global fit indices (CMIN/DF = 1.52–2.40; RMSEA = 0.023–0.037). There was also an improvement of variance clarification by the number of predictors added to the model (R^2^ = 0.005–0.032). Altogether five of 17 predictors in the model showed associations with the weekly amount of walking, all of which had a standardized regression coefficient *β* lower than 0.10. Most associations were found among the motivator predictors (see Appendix A Table A7). 

In the multivariate Model A1 including sociodemographic variables and determinants of the perceived study environment, only aesthetics showed a significant regression coefficient, which was lower than 0.10 (*β* = 0.07). When adding psychological determinants in Model A2, this association disappeared, but three other associations were statistically significant: not living in the university town (*β* = −0.07), bicycle-related crime (*β* = 0.07), and the barrier related to discomfort with study life (*β* = −0.08). All of them showed regression coefficients smaller than *β* < 0.10.

#### 4.2.2. Regression Analyses for Cycling

The regression models for active transportation by cycling showed adequate to good global fit indices (CMIN/DF = 1.52–2.41; RMSEA = 0.023–0.038). There was also an improvement of variance clarification reached by blockwise including the sets of different predictors to the model (R^2^ = 0.05–0.24). Altogether, 12 of 17 predictors showed associations with the weekly amount of cycling, whereas all of the psychological determinants were present. For most predictors, the standardized regression coefficients were considered small to medium size (|0.06| < *r* < |0.38|) (see Appendix A Table A8 and Table A9).

In the multivariate Model B1 including sociodemographic variables and variables of the perceived study environment, five predictors showed a significant regression coefficient, but for two of them it was lower than 0.10. The highest regression coefficient was found for the predictor of resident in the university town (*β* = 0.20), followed by bicycle-related crime (*β* = −0.14), and high automobile traffic (*β* = 0.118). When adding psychological determinants in Model B2, all associations became smaller or, in the case of high automobile traffic, showed a regression coefficient smaller than *β* < 0.10 (*β* = 0.08). The following associations remained with a small to medium regression coefficient: resident in the university town (*β* = 0.14) and bicycle-related crime (*β* = −0.13). While the association with “active transportation: uphill” disappeared, “walking/cycling facilities” were statistically significant but with a regression coefficient smaller than *β* < 0.10. Additionally, three other associations were statistically significant: personal barriers (*β* = −0.24), external barriers (*β* = −0.23), and personal benefits (*β* = 0.13).

## 5. Discussion

Using a socio-ecological approach in a university setting, the present study addresses the question of which conditions of the study environment as well as individual motivators and barriers are related to students’ transport-related walking and cycling. Results show that there were no relevant predictors associated with the amount of transport-related walking: neither sex, age, and place of living nor the study environment or personal motivators and barriers were substantially linked with transport-related walking. In contrast, transport-related cycling was associated with predictors from both depicted conditions of students’ PA behavior, which are important to understand for developing and improving public health interventions: resident in university town, personal benefits, personal barriers, and external barriers relying on individual conditions and high automobile traffic, and bicycle-related crime relying on contextual conditions. Bearing in mind the social-ecological approach of the study, the results reveal multivariate relationships between the level of cycling for transportation and both environmental and individual conditions.

To investigate these relationships, the present study has firstly bundled factors for the perceived study environment regarding the established survey instruments for neighborhood environment NEWS-G and statistical indices of EFA. The same was done for psychological determinants of students for active transportation regarding the study of Shannon et al., (2006) [26]. This procedure has enabled us to link the study environment based upon an adaption of the NEWS-G as well as psychological determinants with the active transport behavior of students, something that has not yet been investigated much in German-speaking countries. So far, only Molina-Gracia et al., (2010) in Spain have used parts of the NEWS besides other aspects to analyze the active commuting of students to university, namely “walking/cycling facilities” (E) [23]. A short version without adaption was used by Peachey and Baller (2015) in a mid-Atlantic undergraduate university with the NEWS-Abbreviate to distinguish environmental characteristics of the living environment between on-campus neighborhoods and off-campus neighborhoods, and to bring this into connection with general PA [54]. While the NEWS assesses the environment of the neighborhood, none of the previous studies used an adaption to access the environment of the study area. Titze et al., (2007) developed a questionnaire based on the literature and focus groups with a special relation to cycling for transportation and the environment along the transport route of students [27]. With the adaption of NEWS-G to the study environment in this study, we wanted to rely on an established survey procedure of the perceived environment and bring it together with the PA-friendliness of the study environment for transport-related PA. The conceptually and empirically derived factors covered areas of the environmental conditions in relation to the study environment: land use mix–access, connectivity, walking/cycling facilities, aesthetics, automobile traffic, and crime safety. The last two factors showed significant correlations for the convenience sample with students’ cycling for transportation, but none showed associations with walking.

That “high automobile traffic” is positively associated with cycling is contrary to the expected result. This association was slightly weakened by adding psychological determinants into the regression model. It seems paradoxical that sampled students’ perceived difficulties, unpleasantness, or insecure feeling when active traveling due to much traffic and noticeable exhaust fumes from cars or buses, is positively related to cycling for transportation. The same contrary effect was found in multinomial regression analysis from Titze et al., (2007) [27] for regular cyclists, who cycle more than three times a week. For irregular cyclists, the perception of traffic did not show any effect at all. One possible explanation is that cyclists are more exposed to the problem and therefore more likely to report it [27]. Further studies should investigate moderation analyses based on a representative sample, whereby psychological determinants should be integrated as moderators between the study environment and active commuting—especially cycling for transportation. 

There is a negative correlation between bicycle-related crime and cycling. Students’ unsafe feeling for leaving even a locked bicycle in the study environment is negatively related to cycling for transportation. This association has repeatedly been reported in the literature [22,25,27]. For example, Rybarczyk and Gallagher (2014) [25] showed that general crime was the strongest barrier for cycling among students and staff of the university, but also bicycle theft was represented under the three most highly ranked barriers. Rybarczyk and Gallagher concluded that the implementation of law enforcement and safe bicycle facility may promote cycling. This was also suggested by Shannon et al., (2006) [26].

Regarding individual conditions, personal barriers showed the strongest associations with cycling. This is in line with the conclusion of Shannon et al., (2006) that reducing barriers to using active transportation modes is likely to be more effective than promoting the benefits of active modes [26]. Further, Rybarczuk and Gallagher (2014) showed that students indicated that any bicycle barrier would cause a decrease in cycling [25]. Our study results reinforce the premise that students’ personal barriers such as physical effort, time effort, and bad mood are negatively related to cycling for transportation. Such personal barriers of time constrains, inconvenience, or physiological discomfort are in accordance with previous findings [21,27]. The same applies to students’ external barriers such as the weather or the time of day. These external inhibiting factors were also found in previous studies [25,26,28]. Nordfjærn et al., (2019) [55] recently showed that those who strongly prioritized convenience tended to use a car for transportation modes. However, the increased awareness of the negative consequences was related to a more use of active transportation and less car use. A positive association with cycling for transportation applies to students’ personal benefits for active transportation such as joy, health, and fitness. This finding is also in line with the positive relation between emotional satisfaction and regular cycling as found by Titze et al., (2007) [27]. It is also in accordance with the association between strong priorities of PA and less public transportation mode use and more use of active transportation found by Nordfjærn et al., (2019) [55]. Overall, the inclusion of the set of psychological factors in the model improved the variance explanation for the cycling behavior of university students, indicating their important role for individual decisions related to transport-related cycling. However, Nordfjærn et al., (2019) showed that besides psychological variables, situational constraints were more important for mode use than psychological variables and are important to consider as well, for example, car ownership or longer walking time [55].

Regarding sociodemographic variables of the sampled students, the association between residence in the university town and cycling was slightly weakened by adding psychological determinants into the regression model but was still significant at medium level. Students’ residence in the university town was positively associated with cycling for transportation. This is in line with the negative impact of distance found in previous studies [21,22,26,28,29] and also with the association between longer walking time from students’ residence to university and the more use of public transportation for less active transportation recently showed by Nordfjærn et al., (2019) [55]. Moreover, Zannat et al., (2020) [56] revealed in terms of city planning the travel time besides the provision of infrastructure as influencing factors for active and public transportation of university students. Furthermore, the factor “personal barriers” of our study, which covers the barrier of time effort, is negatively associated with cycling on a medium level and reinforces this interpretation.

The result that there were no relevant contextual and individual predictors for students’ transport-related walking has already been shown in both the university and community setting. Missing statistical significance for the probability of use of walking for students with environmental incentives was also the case in the results of Rybarczuk and Gallagher (2014) [25]. In communal settings, walking for transportation shows a different association than walking for leisure, which is associated with recreation facilities and aesthetics and green spaces [13,17,36,37]. That the results of this study, which investigated only the domain of active transportation, did not show such correlations, suggests that students were not likely to choose walking as an active mode of transportation for contextual or individual reasons, but rather that it was purely a means of getting from point A to point B. However, in terms of active commuting by students in general, positive associations with the perception of walking and cycling facilities [23], traffic and crime safety [19,21,22,25,27,28], and aesthetic aspects such as the “attractiveness of the surroundings” [27] (p. 72) exist, which could not be shown in this study for walking.

Furthermore, active transportation cannot only be considered in the perspective of promoting PA but also in the perspective of promoting more sustainable modes of transport which in turn has effects on the environment, on the economy, and on the health of people [57]. Some recent studies have dealt with the importance of using sustainable means of transport by the university community [56,57]. The authors of these studies also showed that the mode of transportation is conditioned by particularities of university campuses such as bike share systems [58], tailored and strategically-placed point-of-choice prompts, through which students should switch to active transportation [59], or the distribution of the university scheduled classes on the days of the week [60]. However, in order to make use of the potential to increase cycling among students Grimes and Baker (2020) [58] revealed that bike share systems conditions in university settings must be tailored to the target group, Chim et al., (2020) [60] pointed out that there is only a positive association of university courses on weekdays with more time spent cycling if students cycle to classes anyway, and Irwin (2019) [61] showed that uncontrollable factors for example time, built environment, and weather affected the participation in activities. Thus, just like the results of our study, these findings show that the combination of environmental conditions and personal psychological determinants is important to consider. In addition to tailored measures offered by the university to promote sustainable and active transportation, also competing modes of transportation bring further psychological factors into play. Cruz-Rodriguez et al., (2020) [57] analyzes students’ feelings and emotions provoked by alternative means of transport. In addition to various electric means of transportation, only the use of bicycles showed associations with the possibility of PA, but, for example, the feeling of freedom or getting around quickly in the city or avoiding traffic jams were also present for scooters and motorcycles [57]. Further studies should include deeper psychological backgrounds of transportation choice. To take advantage of the synergies between promoting PA and sustainability, further studies should additionally compare competing modes of transportation such as scooters and motorcycles.

### Strengths and Limitations

Certain limitations must be considered when interpreting the results. Due to the cross-sectional study design, we could not identify causal associations. In addition, the study was conducted in the summertime, which could have an influence on the reported active commuting information due to better weather [19]. Furthermore, regarding the shift toward more female students in the convenience sample of the study, possible sampling bias cannot be excluded. Some studies report a gender difference in favor of male students with regard to the use of bicycles for active transportation [28,30], but other studies did not found different travel patterns between male and female students [23,62]. Agarwal and North (2012) [19] found some gender differences regarding the perception of barriers to cycling. Accordingly, generalizability of the associations would still need to be empirically verified.

The measuring instrument for the study environment was empirically used for the first time. Although the study has attempted to bundle information for both study environment and psychological determinants to better account for psychometric properties of the factors, some variables were measured as single items. For study environment the categories “land use mix–access”, “connectivity,” “general crime”, and “bicycle-related crime” were only covered with one item each. For psychological determinants, the motivator item “avoid air pollution” was considered separately due to content and statistical indices. It is possible that the single items contributed to the absence of associations due to their lower variance. However, it has not been uncommon to include single items in this area of research to date [19,21,26]. Further development is thus needed for measurement procedures. For some areas, the present study provides indications. Our study did form a factor, which dealt with study-related psychological determinants. Furthermore, factors relying on personal benefits, on instrumental extrinsic benefits, and on avoiding air pollution were formed for motivators. Factors for barriers were discomfort with study life, personal barriers, and external barriers. Overall, further surveys in other universities are necessary to concretize and validate the adapted NEWS-G for the study environment as well as to confirm the factors formed.

In addition, the measuring instrument for the study environment captures the self-assessed perception of the students and thus does not provide an objective measure of the survey. This can lead to distortions, for example, as people who frequently walk or cycle outside might perceive traffic more strongly [40]. The importance of perception can only be filtered out and captured through a combination of objective and self-assessed measurement of physical environmental characteristics [41].

Despite the limitations, this study provides some strengths. It tried for the first time to assess not the living environment but the specific study environment with reference to an established survey instrument, so it can be used for campus as well as urban universities. This is important due to the fact that the transfer of results from campus universities is difficult to universities, which are not structured as closed geographical spaces, but the urban university is integrated into urban landscape [24,63].

In addition, referring to socio-ecological approaches could confirm the relationship between transport-related PA and both contextual as well as individual determinants. Further, it provides initial multivariate results on active transportation and its relation to contextual and individual determinants from Germany. Furthermore, since this study differentiated the PA domains into the different modes of transportation, walking and cycling, it could show that the compositional and contextual conditions are different for both modes. So for promoting PA it is important to distinguish between the needs of pedestrians and cyclists [20].

To sum up, in relation to other studies with respect of university students which considered both personal and environmental determinants together in relation with active transportation, the scientific value of the presented study lies in the insights into the contextual conditions of the study environment, the consideration of associated correlates through the factor bundling, and separate information for transport-related cycling and transport-related walking.

## 6. Conclusions

Current findings confirm on a regression-analytical basis the postulated socio-ecological relationships between both contextual as well as individual factors and transport-related cycling, but not with transport-related walking. In total, the students’ amount of cycling a week is positively associated with the students’ residence in the university town, high automobile traffic, and personal benefits such as joy and health, and negatively associated with bicycle-related crime, personal barriers such as physical or time effort, and external barriers such as weather conditions. It should be noted that there might be a partial correlation between “high automobile traffic” and psychological determinants which indicate a moderation role of psychological determinants.

Possible strategies leading to an adequate infrastructure for universities may be the implementation of safe bicycle racks, bicycle routes, or more student residences in town. Additionally, academic training programs that indicate the benefits of transport-related cycling may students help to understand the associations between cycling and health, environment, sports and recreation. This can increase motivation to use the bicycle for transportation and lead to consolidate the bicycle culture in transportation in the university community. Given the current climate change and the increasing physical inactivity of society, a cycling culture can advance alternative means of transportation and thus have positive effects on the economy, environment, and health. As PA is linked with various benefits for health and educational outcomes, the results contribute to the understanding of the correlates of active commuting. This is important especially for university students who are particularly at risk of not fulfilling health-oriented PA recommendations. Therefore, the present study supplements specific knowledge about determinants that are important for developing and improving public health interventions for students in a university setting.

## Figures and Tables

**Table 1:** Overview of the sample as well as of the study variables according to physical activity (dependent variables), perceived study environment, and personal motivators for and barriers to PA.

Variables (Number of Items of the Scale)		*n*	f	f (%)				
Sociodemographic characteristics								
Residence: resident in university town		997	773	77.5				
Residence: not resident in university town			224	22.5				
Gender: female		950	718	24.4				
Gender: male			232	75.6				
	**r/α**	***n***	**M**	**SD**	**min**	**max**	**Skewness**	**Kurtosis**
Age		994	23.40	3.45	18	42	1.37	3.25
Physical activity								
Transportation walking (METh/week) ^1^		993	9.31	9.17	0	40.15	1.55	2.11
Transportation cycling (METh/week) ^2^		991	8.71	12.21	0	51.00	1.63	2.19
Perceived study environment								
Active transportation: uphill (1)		994	2.56	0.99	1	4	−0.02	−1.03
Active transportation: connectivity (1)		996	2.20	0.81	1	4	0.22	−0.47
Active transportation: walking/cycling facilities (2)	0.51	993	1.55	0.57	1	4	1.04	1.06
Aesthetics (3)	0.60	992	2.24	0.59	1	4	0.07	−0.11
High automobile traffic (3)	0.69	989	2.32	0.63	1	4	0.24	−0.26
General crime (1)		997	1.25	0.53	1	4	2.32	5.75
Bicycle-related crime (1)		990	2.19	2.00	1	4	0.47	−0.12
Psychological determinants-Motivators								
Study-related motivator (2)	0.53	971	2.78	0.84	1	4	−0.19	−0.82
Personal benefits (2)	0.42	983	3.00	0.75	1	4	−0.33	−0.65
Instrumental extrinsic (2)	0.43	888	2.55	0.93	1	4	−0.11	−0.97
Avoid air pollution (1)		983	2.69	0.07	1	4	−0.14	−1.01
Psychological determinants-Barriers								
Personal (3)	0.69	975	1.77	0.68	1	4	0.87	0.29
Discomfort with study life (3)	0.77	837	2.11	0.88	1	4	0.41	−0.87
External (2)	0.48	990	2.25	0.82	1	4	0.45	−0.55

^1^ Factor 3.3 for computing MET-minutes for walking. ^2^ Factor 6.0 for computing MET-minutes for cycling.

**Table 2:** Results of the blockwise multivariate regression models A1 (predictors—sociodemographics, and perceived study environment) and A2 (A1 plus motivators and barriers) for the active transportation by walking.

Walk	Model A1		Model A2 (A1 Plus Motivators and Barriers)	
Predictors	*β*	*p*	*β*	*p*
Sociodemographic				
Sex	0.03	0.33	0.02	0.56
Age	0.00	0.92	−0.01	0.88
Resident in university town	−0.06	0.07	−0.07	0.03 *
Perceived study environment				
Active transportation: uphill	−0.01	0.68	0.01	0.87
Active transportation: connectivity	0.03	0.31	0.03	0.41
Active transportation: walking/cycling facilities	0.03	0.40	0.03	0.45
Aesthetics	0.07	0.04 *	0.05	0.16
High automobile traffic	0.01	0.84	0.00	0.96
General crime	−0.02	0.59	−0.02	0.61
Bicycle-related crime	0.06	0.09	0.07	0.04 *
Psychological determinants-Motivators				
Study-related motivator			0.02	0.71
Personal benefits			0.07	0.12
Instrumental extrinsic			−0.06	0.08
Avoid air pollution			0.05	0.22
Psychological determinants-Barriers				
Personal			0.02	0.69
Discomfort with study life			−0.08	0.04 *
External			0.01	0.79
R^2^	0.01		0.03	
ΔR^2^	0.01		0.02	
RMSEA	0.03		0.04	
CFI	0.95		0.95	
CMIN/DF	1.772		2.396	

RMSEA: Root Mean Square Error of Approximation; CFI: Comparative Fit Index; CMIN/DF: ratio of Chi-square (minimum discrepancy) to its Degrees of Freedom; * The probability of error is less than 5%.

**Table 3:** Results of the blockwise multivariate regression models B1 (predictors—sociodemographics, and perceived study environment) and B2 (A1 plus motivators & barriers) for the active transportation by cycling.

Cycle	Model B1		Model B2 (B1 Plus Motivators & Barriers)	
Predictors	*β*	*p*	*β*	*p*
Sociodemographic:				
Sex	−0.06	0.07	−0.04	0.22
Age	0.02	0.52	0.01	0.80
Resident in university town	0.20	<0.01 ***	0.14	<0.01 ***
Perceived study environment:				
Active transportation: uphill	−0.08	0.02 *	0.01	0.70
Active transportation: connectivity	−0.00	0.96	−0.03	0.26
Active transportation: walking/cycling facilities	−0.04	0.22	−0.07	0.02 *
Aesthetics	0.02	0.56	−0.05	0.12
High automobile traffic	0.12	<0.01 ***	0.08	0.01 **
General crime	−0.07	0.04 *	−0.02	0.52
Bicycle-related crime	−0.14	<0.01 ***	−0.13	<0.01 ***
Psychological determinants-Motivators:				
Study-related motivator			−0.01	0.71
Personal benefits			0.13	0.00 **
Instrumental extrinsic			0.06	0.08
Avoid air pollution			0.05	0.11
Psychological determinants-Barriers:				
Personal			−0.24	<0.01 ***
Discomfort with study life			0.04	0.23
External			−0.23	<0.01 ***
R^2^	0.08		0.24	
ΔR^2^	0.03		0.16	
RMSEA	0.03		0.04	
CFI	0.955		0.957	
CMIN/DF	1.767		2.401	

* The probability of error is less than 5%. ** The probability of error is less than or equal to 1%. *** The probability of error is less than or equal to 0.1%.

## Data Availability

The data presented in this study are available on request from the corresponding author.

## Appendix A

**Table A1:** Comparison of the finally selected items and their category bundled by the factor analysis and content fit in this article with the adapted categories of the NEWS-G 1 [44] for the study environment asked in the survey.

Original Items NEWS-G ^1^	Adapted Items of the Survey ^2^	Finally Selected Items and Their Factor (Bolds) ^3^
	(2) Opportunities for walking and cycling:	
(C) Land use mix–access (1 of 7 adopted in the survey)	C7: There are many canyons/hillsides in my study environment that limit the number of routes for getting from place to place.	**Active transportation: uphill** C7: There are many canyons/hillsides in my study environment that limit the number of routes for getting from place to place.
(D) Street connectivity (1 of 6 adopted in the survey)	D5: There are many alternatives (walking/biking) routes for getting from place to place in my study environment. You do not always have to take the same route.	**Active transportation: connectivity** D5: There are many alternatives (walking/biking) routes for getting from place to place in my study environment. You do not always have to take the same route.
(E) Walking/Cycling facilities (2 of 5 adopted in the survey)	E1: There are sidewalks on most of the streets in my study environment. E3: There are bicycle or pedestrian trails in or near my study environment that are easy to get to.	**Active transportation: walking/cycling facilities** E1: There are sidewalks on most of the streets in my study environment. E3: There are bicycle or pedestrian trails in or near my study environment that are easy to get to.
(F) Aesthetics (3 of 6 adopted in the survey)	F1: There are trees along the streets in my study environment. F3: There are many interesting things to look at while walking in my study environment. F5: There is a lot of nature in my study environment that is beautiful to look at (such as landscapes, viewpoints).	**Aesthetics** F1: There are trees along the streets in my study environment. F3: There are many interesting things to look at while walking in my study environment. F5: There is a lot of nature in my study environment that is beautiful to look at (such as landscapes, viewpoints).
	(3) (Traffic) safety:	
(G) Pedestrian/Traffic safety (4 of 8 adopted in the survey; 1 own addition in the survey)	G2: There is so much traffic along nearby streets that it makes it difficult or unpleasant to walk in my study environment G3: The traffic speed in most surrounding streets is normally low (30 km/h or less). G6: There are crosswalks and pedestrian signals to help walkers cross busy streets in my study environment. G8: When walking in my study environment there are a lot of noticeable exhaust fumes (e.g., from cars and buses). New1: Because of the heavy traffic in my study environment, one feels insecure when walking/cycling ^4^.	**High automobile traffic** G2: There is so much traffic along nearby streets that it makes it difficult or unpleasant to walk in my study environment. G8: When walking in my study environment there are a lot of noticeable exhaust fumes (e.g., from cars and buses). New1: Because of the heavy traffic in my study environment, one feels insecure when walking/cycling.^4^
(H) Crime safety (0 of 6 adopted in the survey; 2 own additions in the survey)	New2: I feel unsafe in my study environment during the day due to crime ^4^. New3: It is unsafe to leave even a locked bicycle in my study environment ^5^.	**General crime** New2: I feel unsafe in my study environment during the day due to crime ^4^.
		**Bicycle-related crime** New3: It is unsafe to leave even a locked bicycle in my study environment ^5^.

^1^ Neighborhood Environment Walkability Scale—Germany. ^2^ adapted categories of the NEWS-G for the study environment asked in the survey. ^3^ newly formed by factor analysis and content fit in this article. ^4^ for reasons of condensation, a summarized generalized statement of the respective category of the NEWS-G was newly formed. ^5^ due to previous evidence on the relationship between bicycle thefts and active camouflage transport behavior of students, a new specific item on crime was formed.

**Table A2:** Comparison of the final construct of motivator items newly formed by the factor analysis and content fit in this article.

Original Items	Adapted Items of the Survey	Final Factors ^1^
To compensate for sedentary activities ^2^	Mot5: Active balance between and after courses.	Comfort with study life
To improve my physical performance ^2^	Mot8: Being more efficient for study and work.	
Improvement to health/fitness ^3^	Mot3: Improvement to health/fitness	Personal benefits
Enjoyment gained from current mode ^3^ & Gain sense of enjoyment ^2^	Mot7: Enjoyment in transportation	
Potential to save money ^3^	Mot1: Potential to save money	Instrumental extrinsic benefits
Avoid the need to find parking ^3^ & Unable to obtain parking permit ^3^	Mot2: Avoiding the search for a parking space.	
Personal contribution to reducing air pollution levels ^3^	Mot4: Personal contribution to reducing air pollution levels	Avoid air pollution
Opportunity to socialize ^3^	Mot6: Opportunity to socialize	Not included due to statistical reason

^1^ Newly formed by factor analysis and content fit in this article. ^2^ Own items according to the study “GEDA 2014/2015-EHIS” [44] (p. 118). ^3^ Motivator items (Shannon et al., 2006) [26].

**Table A3:** Comparison of the final construct of barrier items newly formed by the factor analysis and content fit in this article.

Original Items	Adapted Items of the Survey	Final Factors ^1^
I do not feel safe enough to be physically active outdoors alone ^2^ & I feel too fat to exercise	Bar5: I feel uncomfortable participating in university courses after physical exertion.	Discomfort with study life
Necessity of bringing a change of clothes ^3^	Bar6: Necessity of bringing a change of clothes	
Lack of or poor changing/shower facilities at University of Western Australia (UWA) ^3^	Bar7: Lack of or poor changing/shower facilities	
Physical effort involved ^3^	Bar4: Physical effort involved	Personal barriers
Time involved ^3^	Bar11: Time involved	
I lack motivation, I have no interest ^2^ & I am not in the mood ^4^.	Bar12: I lack motivation, I´m not in the mood.	
Weather (rain, wind, or heat) ^3^	Bar1: Inappropriate weather	External barriers
Need to travel to/from UWA at night ^3^	Bar2: Inappropriate time of day.	
-	Bar9: There are too many hills/climbs on the paths ^5^	Not included due to redundancy with study environment
Lack of secure bicycle parking facilities at UWA ^3^	Bar8: Lack of secure bicycle parking facilities	Not included due to redundancy with study environment
Lack of knowledge of quickest and easiest route to UWA ^3^	Bar10: Lack of knowledge of quickest and easiest route	Not included due to statistical reason
Need vehicle for work purposes ^3^	Bar3: Need vehicle for other purposes	Not included due to statistical reason

^1^ Newly formed by factor analysis and content fit in this article. ^2^ Own items according to the study “GEDA 2014/2015-EHIS” [44] (p. 119). ^3^ Barrier items (Shannon et al., 2006) [26]. ^4^ Krämer & Fuchs (2010) [50] (p. 174). ^5^ Supplemented due to site-specific hilly conditions of the University of Tübingen.

**Table A4:** Item’s descriptives (frequencies in %; 1 = totally disagree; 2 = more likely to disagree; 3 = more likely to agree; 4 = totally agree), factor loadings, and communalities (*h*^2^) of the explorative factor analysis (EFA) of perceived study environment.

No.	Item Wording	Descriptives							EFA: Factor Number					
		*n*	1	2	3	4	M	SD	1	2	3	4	5	*h* ^2^
G2	There is so much traffic along nearby streets that it makes it difficult or unpleasant to walk in my study environment.	996	17.77	54.02	25.30	2.91	2.13	0.73	**0.76**	−0.18	−0.03	−0.04	0.03	0.61
G3	The traffic speed in most surrounding streets is normally low (30 km/h or less).	992	13.31	36.49	37.50	12.70	2.50	0.88	**0.54**	−0.18	0.05	−0.26	−0.41	0.56
New1	Because of the heavy traffic in my study environment, one feels insecure when walking/cycling.	991	18.97	49.45	24.22	7.37	2.20	0.83	**0.72**	0.16	−0.05	−0.05	0.26	0.61
G8	When walking in my study environment there are a lot of noticeable exhaust fumes (e.g., from cars and buses).	996	7.93	36.95	39.36	15.76	2.63	0.84	**0.72**	0.03	−0.14	0.05	0.17	0.57
E1	There are sidewalks on most of the streets in my study environment.	994	0.70	4.73	31.09	63.48	3.57	0.62	0.04	**0.83**	0.00	0.08	−0.13	0.71
E3	There are bicycle or pedestrian trails in or near my study environment that are easy to get to.	995	1.21	9.85	43.52	45.43	3.33	0.70	−0.17	**0.73**	0.10	0.28	−0.05	0.66
G6	There are crosswalks and pedestrian signals to help walkers cross busy streets in my study environment.	996	1.31	7.83	46.39	44.48	3.34	0.68	−0.25	**0.57**	0.04	0.01	0.02	0.39
F1	There are trees along the streets in my study environment.	993	3.02	22.96	51.96	22.05	2.93	0.75	−0.01	0.21	**0.70**	−0.32	0.02	0.63
F3	There are many interesting things to look at while walking in my study environment.	997	7.12	36.61	40.52	15.75	2.65	0.83	−0.14	0.04	**0.62**	0.48	0.04	0.64
F5	There is a lot of nature in my study environment that is beautiful to look at (such as landscapes, viewpoints).	996	6.73	32.33	44.78	16.16	2.70	0.82	−0.08	−0.06	**0.83**	0.14	−0.05	0.73
C7	There are many canyons/hillsides in my study environment that limit the number of routes for getting from place to place.	994	15.59	33.10	30.68	20.62	2.56	0.99	0.05	−0.21	0.18	**−0.67**	0.05	0.53
D5	There are many alternatives (walking/biking) routes for getting from place to place in my study environment. You do not always have to take the same route.	996	5.22	28.71	46.79	19.28	2.80	0.81	−0.01	0.08	0.21	**0.67**	0.01	0.51
New2	I feel unsafe in my study environment during the day due to crime.	997	79.34	17.35	2.71	0.60	1.25	0.53	0.10	−0.15	0.01	0.00	**0.71**	0.54
New3	It is unsafe to leave even a locked bicycle in my study environment.	990	17.88	52.83	21.62	7.68	2.19	0.82	0.18	0.00	−0.01	−0.07	**0.75**	0.60

The backgrounds highlight the largest part of the frequency distribution of the scale in each case. The bolds highlight the largest rotated factor loading in each case.

**Table A5:** Item’s descriptives (frequencies in %; 1 = totally disagree; 2 = more likely to disagree; 3 = more likely to agree; 4 = totally agree), factor loadings, and communalities (*h*^2^) of the explorative factor analysis (EFA) of motivator items.

No.	Item Wording	Descriptives							EFA: Factor Number		
		*n*	1	2	3	4	M	SD	1	2	*h* ^2^
Mot8	Being more efficient for study and work.	977	15.05	34.60	28.05	22.31	2.58	1.00	**0.79**	0.11	0.63
Mot7	Enjoyment in transportation	987	6.48	29.08	33.13	31.31	2.89	0.92	**0.79**	−0.04	0.62
Mot5	Active balance between and after courses.	989	7.08	23.05	34.48	35.39	2.98	0.93	**0.79**	0.10	0.63
Mot3	Improvement to health/fitness	989	3.24	22.04	36.10	38.62	3.10	0.85	**0.71**	0.33	0.61
Mot1	Potential to save money	986	24.04	31.95	24.85	19.17	2.39	1.10	0.06	**0.81**	0.66
Mot4	Personal contribution to reducing air pollution levels	983	12.31	31.03	32.25	24.42	2.69	0.95	0.48	**0.48**	0.46
Mot2	Avoiding the search for a parking space	893	22.06	16.46	28.78	32.70	2.72	1.14	0.07	**0.81**	0.66

The backgrounds highlight the largest part of the frequency distribution of the scale in each case. The bolds highlight the largest rotated factor loading in each case.

**Table A6:** Item’s descriptives (frequencies in %; 1 = totally disagree; 2 = more likely to disagree; 3 = more likely to agree; 4 = totally agree), factor loadings and communalities (*h*^2^) of the explorative factor analysis (EFA) of barrier items.

No.	Item Wording	Descriptives						EFA: Factor Number				
		*n*	1	2	3	4	M	SD	1	2	3	*h* ^2^
Bar4	Physical effort involved	992	49.29	39.62	8.57	2.52	1.64	0.74	**0.69**	0.33	0.01	0.58
Bar11	Time involved.	985	41.32	31.68	17.36	9.64	1.95	0.99	**0.68**	0.09	0.31	0.57
Bar12	I lack motivation, I’m not in the mood.	990	51.21	32.42	11.72	4.65	1.70	0.85	**0.82**	0.11	0.08	0.69
Bar10	Lack of knowledge of quickest and easiest route.	932	61.48	20.39	12.12	6.01	1.63	0.92	**0.51**	0.16	0.27	0.35
Bar5	I feel uncomfortable participating in university courses after physical exertion.	982	37.17	33.20	18.84	10.79	2.03	1.00	0.31	**0.75**	0.10	0.68
Bar6	Necessity of bringing a change of clothes	934	37.37	27.30	20.24	15.10	2.13	1.08	0.18	**0.82**	0.20	0.74
Bar7	Lack of or poor changing/shower facilities	861	36.82	24.16	20.56	18.47	2.21	1.13	0.09	**0.80**	0.15	0.68
Bar1	Inappropriate weather	995	12.56	43.12	25.73	18.59	2.50	0.94	0.22	0.23	**0.67**	0.55
Bar2	Inappropriate time of day.	991	38.14	32.90	19.07	9.89	2.01	0.98	0.34	0.06	**0.71**	0.62
Bar3	Need vehicle for other purposes	875	58.63	22.06	14.17	5.14	1.66	0.90	−0.01	0.12	**0.72**	0.53

The backgrounds highlight the largest part of the frequency distribution of the scale in each case. The bolds highlight the largest rotated factor loading in each case.

**Table A7:** Results (inter-item-correlation-coefficients r, β-coefficients and significant *p*-value) for the bivariate correlation and multivariate regression models A1 (sociodemographics and perceived study environment) and A2 (A1 plus Motivators & Barriers) for the transportation mode “walk”.

Walk	Bivariate Correlation with Walking (MET/Week)		Model A1		Model A2 (A1 Plus Motivators & Barriers)	
Predictors	*r*	*p*	*β*	*p*	*β*	*p*
Sociodemographic						
Sex	0.03	0.39	0.03	0.33	0.02	0.56
Age	0.01	0.74	0.00	0.92	−0.01	0.88
Resident in university town	−0.06	0.06	−0.06	0.07	−0.07	0.03 *
Perceived study environment						
Active transportation: uphill	−0.03	0.39	−0.01	0.68	0.01	0.87
Active transportation: connectivity	−0.06	0.07	0.03	0.31	0.03	0.41
Active transportation: walking/cycling facilities	−0.05	0.13	0.03	0.40	0.03	0.45
Aesthetics	−0.08	0.02 *	0.07	0.04 *	0.05	0.16
High automobile traffic	−0.00	0.94	0.01	0.84	0.00	0.96
General crime	0.00	0.99	−0.02	0.59	−0.02	0.61
Bicycle-related crime	0.05	0.12	0.06	0.09	0.07	0.04 *
Psychological determinants-Motivators						
Study-related motivator	0.08	0.02 *			0.02	0.71
Personal benefits	0.10	0.00 **			0.07	0.12
Instrumental extrinsic	−0.02	0.59			−0.06	0.08
Avoid air pollution	0.07	0.03 *			0.05	0.22
Psychological determinants-Barriers						
Personal	−0.05	0.12			0.02	0.69
Discomfort with study life	−0.09	0.01 *			−0.08	0.04 *
External	−0.03	0.40			0.01	0.79
R^2^			0.01		0.03	
ΔR^2^			0.01		0.02	
RMSEA			0.03		0.04	
CFI			0.95		0.95	
CMIN/DF			1.772		2.396	

* The probability of error is less than 5%. ** The probability of error is less than or equal to 1%.

**Table A8:** Results (inter-item-correlation-coefficients r, β-coefficients and significant *p*-value) for the bivariate correlation and multivariate regression models B1 (sociodemographics and perceived study environment) and B2 (B1 plus Motivators & Barriers) for the transportation mode “cycle”.

Cycle	Bivariate Correlation with Cycling (MET/Week)		Model B1		Model B2 (B1 Plus Motivators & Barriers)		
Predictors	*r*	*p*	*β*	*p*	*β*		*p*
Sociodemographic:							
Sex	−0.05	0.16	−0.06	0.07	−0.04		0.22
Age	0.02	0.50	0.02	0.52	0.01		0.80
Resident in university town	0.21	<0.01 ***	0.20	<0.01 ***	0.14		<0.01 ***
Perceived study environment:							
Active transportation: uphill	−0.06	0.045 *	−0.08	0.02 *	0.01		0.70
Active transportation: connectivity	0.01	0.83	−0.00	0.96	−0.03		0.26
Active transportation: walking/cycling facilities	0.03	0.32	−0.04	0.22	−0.07		0.02 *
Aesthetics	0.00	0.998	0.02	0.56	−0.05		0.12
High automobile traffic	0.06	0.05 *	0.12	<0.01 ***	0.08		0.01 **
General crime	−0.10	0.00 **	−0.07	0.04 *	−0.02		0.52
Bicycle-related crime	−0.14	<0.01 ***	−0.14	<0.01 ***	−0.13		<0.01 ***
Psychological determinants-Motivators:							
Study-related motivator	0.17	<0.01 ***			−0.01		0.71
Personal benefits	0.25	<0.01 ***			0.13		0.00 **
Instrumental extrinsic	0.08	0.015 *			0.06		0.08
Avoid air pollution	0.18	<0.01 ***			0.05		0.11
Psychological determinants-Barriers:							
Personal	−0.38	<0.01 ***			−0.24		<0.01 ***
Discomfort with study life	−0.16	<0.01 ***			0.04		0.23
External	−0.35	<0.01 ***			−0.23		<0.01 ***
R^2^				0.08		0.24	
ΔR^2^				0.03		0.16	
RMSEA				0.03		0.04	
CFI				0.955		0.957	
CMIN/DF				1.767		2.401	

* The probability of error is less than 5%. ** The probability of error is less than or equal to 1%. *** The probability of error is less than or equal to 0.1%.

**Table A9:** Results (inter-item-correlation-coefficients r, and significant *p*-value) for the bivariate correlation between all variables of the regression models.

			1	2	3	4	5	6	7	8	9	10	11	12	13	14	15	16	17
**1**	**Sex**	*r*	1																
		*p*																	
		*n*	950																
**2**	**Age**	*r*	−0.04	1															
		*p*	0.19																
		*n*	947	994															
**3**	**Resident in university town**	*r*	0.050	−0.02	1														
		*p*	0.13	0.50															
		*n*	950	994	997														
**4**	**Active transportation: uphill**	*r*	−0.00	−0.07 *	0.05	1													
		*p*	0.98	0.03	0.12														
		*n*	948	991	994	994													
**5**	**Active transportation: connectivity**	*r*	0.00	−0.04	0.02	0.18 **	1												
		*p*	1.00	0.23	0.48	0.00													
		*n*	949	993	996	993	996												
**6**	**Active transportation: walking/Cycling facilities**	*r*	0.03	0.05	0.04	0.25 **	0.21 **	1											
		*p*	0.43	0.14	0.22	0.00	0.00												
		*n*	946	990	993	990	992	993											
**7**	**Aesthetics**	*r*	0.00	−0.05	0.02	0.03	0.21 **	0.17 **	1										
		*p*	0.89	0.14	0.63	0.37	0.00	0.00											
		*n*	945	989	992	989	991	988	992										
**8**	**High automobile traffic**	*r*	0.08 *	0.02	−0.01	0.09 **	0.09**	0.25 **	0.18 **	1									
		*p*	0.02	0.45	0.69	0.00	0.00	0.00	0.00										
		*n*	942	986	989	986	988	985	984	989									
**9**	**General crime**	*r*	0.07 *	−0.01	−0.10 **	0.03	−0.01	0.16 **	0.03	0.19 **	1								
		*p*	0.03	0.74	0.00	0.43	0.78	0.00	0.29	0.00									
		*n*	950	994	997	994	996	993	992	989	997								
**10**	**Bicycle-related crime**	*r*	0.03	0.06	−0.06	0.09 **	−0.01	0.09 **	0.05	0.24 **	0.26 **	1							
		*p*	0.41	0.09	0.07	0.01	0.86	0.00	0.13	0.00	0.00								
		*n*	943	988	990	987	989	987	985	983	990	990							
**11**	**Study-related motivator**	*r*	0.12 **	−0.01	0.03	−0.03	−0.04	−0.06	−0.14 **	0.13 **	0.04	0.01	1						
		*p*	0.00	0.55	0.41	0.33	0.19	0.08	0.00	0.00	0.19	0.73							
		*n*	924	968	971	968	970	967	966	963	971	965	971						
**12**	**Personal benefits**	*r*	0.11 **	0.09 **	0.06 *	−0.08 *	−0.10 **	−0.05	−0.17 **	0.09 **	−0.01	−0.02	0.66**	1					
		*p*	0.00	0.01	0.05	0.01	0.00	0.16	0.00	0.00	0.80	0.58	0.00						
		*n*	936	980	983	980	982	979	979	975	983	976	962	983					
**13**	**Instrumental extrinsic benefits**	*r*	0.04	−0.01	−0.05	−0.04	−0.10 **	−0.03	−0.07 *	0.10 **	0.09 **	−0.01	0.22 **	0.21 **	1				
		*p*	0.31	0.73	0.14	0.270	0.00	0.32	0.04	0.01	0.01	0.89	0.00	0.00					
		*n*	846	885	888	885	887	884	883	881	888	883	868	877	888				
**14**	**Avoid air pollution**	*r*	0.08 *	−0.01	0.00	−0.06 *	−0.10 **	−0.06	−0.13 **	0.12 **	−0.01	−0.06	0.36 **	0.44 **	0.33 **	1			
		*p*	0.02	0.86	0.92	0.05	0.00	0.06	0.00	0.00	0.83	0.06	0.00	0.00	0.00				
		*n*	936	980	983	980	982	979	978	976	983	977	961	971	881	983			
**15**	**Personal barrier**	*r*	0.04	−0.04	−0.16 **	0.23 **	0.11 **	0.15 **	0.12 **	0.05	0.12 **	0.07 *	−0.26 **	−0.33 **	−0.02	−0.162 **	1		
		*p*	0.22	0.27	0.00	0.00	0.00	0.00	0.00	0.10	0.00	0.03	0.00	0.00	0.60	0.000			
		*n*	929	972	975	972	974	971	970	967	975	969	952	962	870	964	975		
**16**	**Discomfort with study life**	*r*	−0.00	−0.03	−0.08 *	0.26 **	0.12 **	0.11 **	0.12 **	0.18 **	0.05	0.17 **	−0.06	−0.08 *	0.01	−0.076 *	0.46 **	1	
		*p*	0.98	0.42	0.02	0.00	0.00	0.00	0.00	0.00	0.18	0.00	0.12	0.03	0.77	0.029	0.00		
		*n*	798	834	837	834	836	833	832	830	837	832	823	826	756	828	825	837	
**17**	**External barrier**	*r*	0.13 **	−0.02	−009 **	0.18 **	0.08 **	0.13 **	0.09 **	0.11 **	0.16 **	0.08 *	−0.09 **	−0.14 **	0.02	−0.118 **	0.44**	0.38 **	1
		*p*	0.00	0.48	0.00	0.00	0.01	0.00	0.00	0.00	0.00	0.02	0.01	0.00	0.59	0.000	0.00	0.000	
		*n*	943	987	990	987	989	986	985	982	990	983	965	976	882	977	969	832	990

* The probability of error is less than 5%. ** The probability of error is less than or equal to 1%.
